# Assessment of residents’ total environmental exposure to heavy metals in China

**DOI:** 10.1038/s41598-019-52649-w

**Published:** 2019-11-08

**Authors:** Xiuge Zhao, Zhenglei Li, Danlu Wang, Ji Li, Bin Zou, Yan Tao, Limin Lei, Feiyang Qiao, Ju Huang

**Affiliations:** 1 State Key Laboratory of Environmental Criteria and Risk Assessment, Chinese Research Academy of Environmental Sciences, Beijing, 100012 China; 2 Key Laboratory of Environmental Pollution Prediction and Control of Gansu Province, College of Earth and Environmental Sciences,Lanzhou University, Lanzhou, 730000 China; 3 School of Geosciences and Info-Physics, Central South University, Changsha, 410086 China

**Keywords:** Environmental impact, Environmental impact, Environmental impact, Environmental impact

## Abstract

Heavy metal pollution in the air, water, and soil has attracted substantial interest recently; however, assessment of the total human environmental exposure remains limited. Therefore, determining the total human environmental exposure is imperative for the management and control of heavy metal pollution. This study assessed the total environmental exposure levels of heavy metals as well as the exposure contributions of air, water, and soil, focusing on Hg, Cd, As, Pb, and Cr. Data from 3,855 volunteers from the cities of Taiyuan, Dalian, Shanghai, Wuhan, Chengdu, and Lanzhou allowed for comparison of the exposures in urban and rural areas. The levels of total human environmental exposure of Hg, Cd, As, Pb, and Cr were 1.82 × 10^−6^ mg/(kg·d), 1.58 × 10^−6^ mg/(kg·d), 3.87 × 10^−5^ mg/(kg·d), 1.79 × 10^−5^ and 7.47 × 10^−5^ mg/(kg·d), respectively. There were regional, urban-rural, sex, and age differences in the levels of heavy metal exposure. Water pollution was determined to be the largest contributor to heavy metal exposure, accounting for 97.87%, 92.50%, 80.51%, 76.16% and 79.46% of the Hg, Cd, As, Pb, and Cr, followed by air and soil pollution. These results can provide data to inform environmental protection policies and identify the priority pollutants that can help identify and prevent health risks due to overexposure to these heavy metal pollutants.

## Introduction

Massive amounts of heavy metals from persistent anthropogenic activities in urbanization and industrialization are released into the atmosphere, water, soil, and crops, threatening ecological conditions and human health^1,2^. Metal pollution is a severe problem and has been described as a “chemical bomb” in China due to its ubiquity, toxicity at trace levels, bioaccumulation, and persistence^3,4^. Chromium (Cr) is an essential trace element involved in basal metabolism, but too much in the body may lead to negative effects, partly because Cr is a known human carcinogen and anaphylactogen^5,6^. Mercury (Hg) can accumulate in adipose tissue and cause damage to the nervous and immune systems^7–9^. Arsenic (As) and cadmium (Cd) are known to increase the risks of lung cancer and renal carcinoma^10,11^. Long-term exposure to lead (Pb) can be harmful to the circulatory and central nervous systems^12^. Thus, a full assessment of the total exposure to heavy metals can help increase the general health of Chinese residents.

Total human environmental exposure seeks to estimate the exposure of the population to particular environmental pollutants through multiple exposure media simultaneously^13,14^. Total human environmental exposure can provide exposure levels of the general public, with a known precision and accuracy, through all possible environmental media, including air, drinking water, food, and skin contact^13,14^. The first total exposure study was performed in America in 1979 and at determining the exposure-response relationship of volatile organic compounds through different media^13,14^. The results of this study were used to establish the Total Risk Integrated Methodology (TRIM) and correlative models^15,16^. American and Japanese scholars each simulated the exposure levels and media contribution by Monte-Carlo models and found that drinking water was the main exposure medium in America, with the exception of diet for arsenic, while soil was the largest contributor in Japan^17,18^. In 2010, a study in England found that soil was the main exposure media of Cr^19^, while drinking water was found to be the source of many trace metals, including Hg, Pb, Cd, Cr, Se, As, and Ni, in Iran^20^.

Total human environmental exposure studies are unique because humans are the centre of these studies^13^. This is significantly different from traditional health risk assessment methods, which generally ignore human activity patterns. Traditional health risk assessments have failed to attach importance to variations of the microenvironment (such as time spent indoors or outdoors)^14^. It is easy for total human environmental exposure to differentiate between exposure levels and exposure contributions from different environmental media. However, no systematic research on the total human environmental exposure of heavy metals covering regional, seasonal, urban-rural, sex, and age differences has been conducted in China. Therefore, it is necessary and important to study the total human environmental exposure of heavy metals to identify exposure levels and, consequently, to better understand how to reduce the exposure to these pollutants for Chinese residents. This study will finally provide a scientific basis for determining and implementing environmental health benchmarks and the order of priority for cleaning up these pollutants. Taiyuan, Dalian, Shanghai, Wuhan, Chengdu and Lanzhou were chosen as cities representatives of a typical inland coal-smoke city, a coastal industrial city, a first-tier and business-oriented metropolis, a port city along a river, an industrial city located in the Sichuan Basin and an inland city polluted by dust, respectively. Urban and rural areas were selected to identify the total exposure levels and contributions of different media for Hg, Cr, As, Pb, and Cd for residents in these areas.

Considering that the assessment of the total human environmental exposure remains limited and that it can identify the dominant source of human exposure, which is related to human activity patterns, this study aimed to assess the total human environmental exposure level of heavy metals (Hg, Cr, As, Pb, and Cd) and the exposure contribution of air, water, and soil based on data collected from residents in Taiyuan, Dalian, Shanghai, Wuhan, Chengdu, and Lanzhou while allowing for the comparison of the exposure in urban and rural areas. The output of the study was expected to contribute to the drafting and revision of correlative environmental health standards and criteria, fill gaps in total human environmental exposure data, and ascertain the key point for public health.

## Results and Discussion

### Heavy metal concentrations

**Figure 1:**
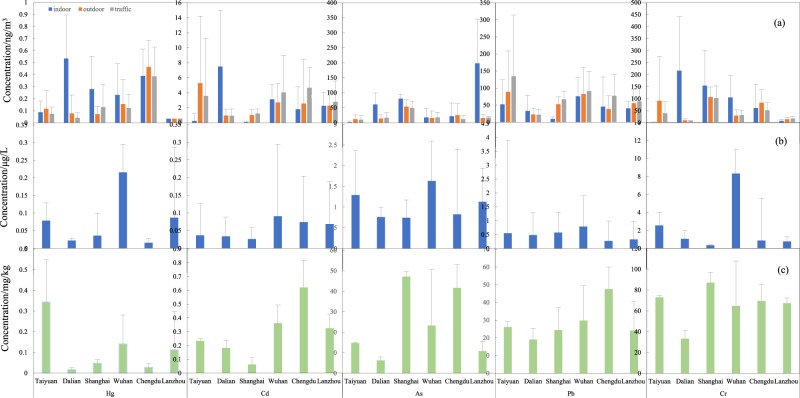
The concentration of Hg, Cd, As, Pb, and Cr in the (**a**) air, (**b**) water, and (**c**) soil in the different areas tested.

The basic statistics of the heavy metal concentrations (Hg, Cr, As, Pb, and Cd) in air, water, and soil in the six chosen cities are shown in Fig. 1. The different cities have different levels of each of the heavy metal pollutants. There are differences in heavy metal concentrations in urban and rural areas as well as in the three environmental media tested (air, water, and soil). There are regional differences with regard to regulations for specific metals. The highest concentrations of Hg from indoor, outdoor, and traffic air samples were in Chengdu, while the lowest concentrations were found in Lanzhou. The concentration of Hg from water was highest in Wuhan and lowest in Chengdu, while the concentration in the soil was highest in Taiyuan and lowest in Dalian. The concentrations of Cd from indoor air and water were the highest in Wuhan, and the concentrations from traffic air and soil were the highest in Chengdu. The concentration of Cd from outdoor air was the highest in Taiyuan. Of the six cities in this study, Shanghai had the lowest concentrations of Cd in the different media, except water. The concentrations of As from indoor air, outdoor air, traffic air, water, and soil were highest in Lanzhou, Shanghai, Shanghai, Taiyuan and Chengdu and lowest in Taiyuan, Wuhan, Chengdu, Dalian, and Dalian, respectively. The Pb concentrations of indoor and traffic air were the highest in Taiyuan. The Pb concentrations in outdoor air and water were the highest in Wuhan, while the largest concentration in soil was in Chengdu. The lead concentrations of indoor air and soil were lowest in Shanghai. The concentrations of Pb from outdoor and traffic air were the lowest in Dalian, while the concentration of Pb in the water was the lowest in Chengdu. The concentrations of Cr from indoor air, outdoor air, traffic air, water, and soil were highest in Chengdu, Taiyuan, Shanghai, Wuhan and Shanghai, respectively. The lowest Cr concentrations from outdoor air, traffic air and soil were found in Dalian, while the lowest concentration of Cr from indoor air was found in Taiyuan, and the concentration of Cr in water was the lowest in Shanghai. This study also showed that the concentration of Cr was the highest among the heavy metals from soil in these cities, consistent with an Iranian study that showed this as well^20^.

### Exposure levels of heavy metals

The total exposure levels of the five heavy metals (Table 1) were ranked in the order of Cr > As > Pb > Hg > Cd, averaging 7.47 × 10^−5^ mg/(kg·d), 3.87 × 10^−5^ mg/(kg·d), 1.79 × 10^−5^ mg/(kg·d), 1.82 × 10^−6^ mg/(kg·d) and 1.58 × 10^−6^ mg/(kg·d), respectively. There were significant regional and seasonal differences in the six cities for Hg, Cd, As, Pb, and Cr (p < 0.01). The highest exposure levels of Hg, Cd, As, and Pb were found in Wuhan and the lowest were found in Dalian. Cr was found to have the highest exposure levels in Wuhan and the lowest in Shanghai. The ratio of the exposure levels in the highest exposure city to the levels in the lowest exposure city for Hg, Cd, As, Pb, and Cr were 14.12, 4.69, 3.18, 2.33, 11.68, respectively. The level of total human environmental exposure in this study is lower than the concentration of inorganic As reported for the population between 16 and 59 years old in a 2002 American study^18^. The total human environmental exposure of As, Cd, Cr, and Pb in this study are higher than the results of a French study involving children between 3 and 6 years old^21^.

**Table 1:** Exposure levels of Hg, Cd, As, Pb and Cr (mg/(kg·d)).

Exposure medium			Total		Taiyuan		Dalian		Shanghai		Wuhan		Chengdu		Lanzhou	
			Mean	S.D	Mean	S.D	Mean	S.D	Mean	S.D	Mean	S.D	Mean	S.D	Mean	S.D
Hg	Total		1.82E-06	2.21E-06	1.64E-06	8.44E-07	3.93E-07	1.89E-07	7.55E-07	4.84E-07	5.55E-06	2.61E-06	5.47E-07	2.79E-07	2.01E-06	1.80E-06
	Air	Total	2.18E-09	1.58E-09	8.29E-10	3.25E-10	3.64E-09	9.08E-10	2.54E-09	1.41E-09	2.31E-09	1.08E-09	3.82E-09	7.59E-10	3.02E-10	4.37E-11
		Indoor	1.91E-09	1.47E-09	6.47E-10	2.89E-10	3.47E-09	8.84E-10	2.40E-09	1.39E-09	2.08E-09	1.10E-09	2.98E-09	7.99E-10	2.33E-10	4.21E-11
		Outdoor	2.16E-10	2.94E-10	1.55E-10	1.04E-10	1.47E-10	9.59E-11	6.77E-11	2.01E-11	1.94E-10	1.19E-10	6.87E-10	4.60E-10	5.39E-11	2.61E-11
		Traffic	5.29E-11	5.67E-11	2.76E-11	2.51E-11	1.70E-11	9.24E-12	6.57E-11	3.62E-11	4.48E-11	2.31E-11	1.53E-10	5.67E-11	1.52E-11	9.39E-12
	Water		1.80E-06	2.20E-06	1.63E-06	8.42E-07	3.84E-07	1.88E-07	7.46E-07	4.86E-07	5.51E-06	2.61E-06	5.31E-07	2.83E-07	1.99E-06	1.80E-06
	Soil		1.76E-08	3.94E-08	1.89E-08	4.63E-08	4.81E-09	6.80E-09	6.39E-09	1.45E-08	4.15E-08	6.72E-08	1.22E-08	1.91E-08	2.11E-08	3.28E-08
Cd	Total		1.58E-06	1.65E-06	7.86E-07	6.88E-07	6.10E-07	2.89E-07	7.27E-07	5.31E-07	2.86E-06	2.24E-06	2.66E-06	1.67E-06	1.86E-06	1.64E-06
	Air	Total	2.18E-08	1.75E-08	9.62E-09	4.93E-09	4.99E-08	1.68E-08	2.22E-09	4.72E-10	2.81E-08	6.71E-09	2.09E-08	1.11E-08	2.08E-08	6.60E-09
		Indoor	1.70E-08	1.78E-08	1.27E-09	2.50E-09	4.79E-08	1.68E-08	7.57E-10	3.10E-10	2.29E-08	5.56E-09	1.51E-08	8.27E-09	1.58E-08	6.64E-09
		Outdoor	3.55E-09	3.61E-09	6.97E-09	4.39E-09	1.64E-09	6.78E-10	8.63E-10	3.49E-10	3.65E-09	2.28E-09	3.94E-09	5.07E-09	3.71E-09	1.75E-09
		Traffic	1.18E-09	1.13E-09	1.38E-09	1.74E-09	3.74E-10	1.92E-10	5.96E-10	2.95E-10	1.54E-09	1.11E-09	1.84E-09	6.81E-10	1.30E-09	1.01E-09
	Water		1.49E-06	1.61E-06	7.44E-07	6.79E-07	5.10E-07	2.64E-07	7.18E-07	5.34E-07	2.75E-06	2.26E-06	2.49E-06	1.55E-06	1.74E-06	1.61E-06
	Soil		7.05E-08	1.39E-07	3.19E-08	7.68E-08	5.00E-08	7.07E-08	6.40E-09	1.51E-08	7.82E-08	1.27E-07	1.50E-07	2.31E-07	1.05E-07	1.48E-07
As	Total		3.87E-05	3.45E-05	3.19E-05	1.85E-05	1.82E-05	9.18E-06	2.33E-05	1.86E-05	5.79E-05	3.57E-05	5.57E-05	5.57E-05	4.45E-05	2.60E-05
	Air	Total	5.22E-07	5.57E-07	3.19E-08	1.94E-08	4.43E-07	8.76E-08	6.92E-07	1.12E-07	1.54E-07	8.90E-08	3.20E-07	3.55E-07	1.46E-06	5.34E-07
		Indoor	4.84E-07	5.53E-07	1.28E-08	1.37E-08	4.10E-07	8.45E-08	6.22E-07	1.06E-07	1.25E-07	8.15E-08	2.71E-07	3.53E-07	1.43E-06	5.36E-07
		Outdoor	2.95E-08	2.20E-08	1.56E-08	8.35E-09	2.63E-08	1.13E-08	4.60E-08	1.80E-08	2.25E-08	1.68E-08	4.39E-08	3.56E-08	2.53E-08	1.19E-08
		Traffic	8.81E-09	8.96E-09	3.44E-09	3.13E-09	6.89E-09	3.68E-09	2.42E-08	1.16E-08	6.75E-09	4.47E-09	5.15E-09	3.54E-09	7.79E-09	4.90E-09
	Water		2.57E-05	1.98E-05	2.61E-05	1.24E-05	1.32E-05	7.10E-06	1.48E-05	7.66E-06	4.05E-05	2.68E-05	2.59E-05	1.63E-05	3.24E-05	2.37E-05
	Soil		1.25E-05	2.71E-05	5.81E-06	1.47E-05	4.48E-06	6.56E-06	7.86E-06	1.80E-05	1.72E-05	3.08E-05	2.95E-05	4.75E-05	1.06E-05	1.54E-05
Pb	Total		1.79E-05	1.87E-05	1.43E-05	2.40E-05	1.23E-05	7.27E-06	1.39E-05	8.62E-06	2.87E-05	1.77E-05	2.43E-05	2.55E-05	1.47E-05	1.32E-05
	Air	Total	4.46E-07	2.64E-07	5.69E-07	1.76E-07	2.81E-07	9.18E-08	1.51E-07	2.93E-08	7.02E-07	1.33E-07	5.52E-07	3.85E-07	4.00E-07	1.13E-07
		Indoor	3.38E-07	2.41E-07	4.01E-07	1.38E-07	2.28E-07	8.03E-08	7.40E-08	1.63E-08	5.55E-07	1.23E-07	4.73E-07	3.91E-07	2.85E-07	8.66E-08
		Outdoor	7.64E-08	5.71E-08	1.16E-07	7.08E-08	4.26E-08	1.97E-08	4.45E-08	1.68E-08	1.12E-07	6.63E-08	4.87E-08	3.38E-08	8.72E-08	4.59E-08
		Traffic	3.16E-08	2.76E-08	5.20E-08	4.74E-08	9.95E-09	6.67E-09	3.27E-08	1.60E-08	3.42E-08	1.74E-08	3.02E-08	1.18E-08	2.82E-08	2.17E-08
	Water		1.09E-05	1.25E-05	1.08E-05	2.23E-05	7.60E-06	4.77E-06	1.09E-05	5.23E-06	1.96E-05	1.25E-05	8.61E-06	4.80E-06	8.27E-06	8.77E-06
	Soil		6.59E-06	1.32E-05	2.96E-06	7.11E-06	4.38E-06	6.21E-06	2.90E-06	6.63E-06	8.38E-06	1.36E-05	1.52E-05	2.35E-05	6.02E-06	8.59E-06
Cr	Total		7.47E-05	9.35E-05	6.30E-05	3.91E-05	2.76E-05	1.98E-05	1.97E-05	2.71E-05	2.30E-04	1.05E-04	6.99E-05	9.26E-05	4.10E-05	3.06E-05
	Air	Total	7.61E-07	6.20E-07	1.51E-07	9.90E-08	1.46E-06	3.64E-07	1.31E-06	2.47E-07	9.10E-07	4.15E-07	8.18E-07	5.31E-07	8.56E-08	2.80E-08
		Indoor	6.69E-07	6.27E-07	1.00E-08	3.32E-09	1.44E-06	3.62E-07	1.17E-06	2.32E-07	8.60E-07	4.21E-07	6.49E-07	5.47E-07	5.46E-08	2.25E-08
		Outdoor	7.45E-08	8.18E-08	1.26E-07	1.07E-07	1.77E-08	7.51E-09	9.32E-08	2.93E-08	3.74E-08	2.12E-08	1.49E-07	1.02E-07	2.28E-08	1.23E-08
		Traffic	1.76E-08	1.95E-08	1.53E-08	1.90E-08	3.10E-09	1.56E-09	4.89E-08	2.34E-08	1.19E-08	6.29E-09	2.04E-08	7.51E-09	8.17E-09	5.15E-09
	Water		5.89E-05	8.67E-05	5.37E-05	2.91E-05	1.66E-05	1.00E-05	6.61E-06	2.39E-06	2.09E-04	1.01E-04	5.10E-05	8.13E-05	2.03E-05	1.23E-05
	Soil		1.50E-05	2.68E-05	9.13E-06	2.20E-05	9.57E-06	1.35E-05	1.18E-05	2.66E-05	2.09E-05	3.41E-05	1.80E-05	2.79E-05	2.06E-05	2.91E-05

For every heavy metal tested, urban-rural residence, age, and sex were influencing factors on the total human environmental exposure of the volunteers (Table 2). It is significant that the total exposure had an urban-rural difference (p < 0.05), illustrating that the total exposure of rural residents to Cr, As, Pb, and Cd was higher than the total exposure in urban areas. The ratio of exposure levels in urban areas and rural areas for Hg, Cd, As, Pb, and Cr averaged 1.37, 0.85, 0.61, 0.46, and 0.59, respectively. For Hg, Cd, As, Pb, and Cr, the exposure levels of men were slightly higher than those of women. The exposure levels of Hg and As of men and women were significantly different (p < 0.05) because of the larger comprehensive exposure coefficient for men. The ratios of exposure levels for men and women to Hg, Cd, As, Pb, and Cr were 1.01, 1.00, 1.05, 1.01, and 1.03, respectively. The exposure level of the population between 18 and 44 years old was the lowest overall, while the highest exposure to Hg, Cd, Pb, and Cr was observed in residents over 60 years old, and the highest exposure level of As was in the population aged between 45 and 59 years old. Our results are consistent with the total exposure study performed in Iran, which showed that the population over 55 years old were exposed to higher levels of heavy metals through a combination of air, drinking water, and soil^20^.

**Table 2:** Exposure levels of Hg, Cd, As, Pb and Cr for different areas, sex, and age groups (mg/(kg·d)).

Metal		Hg	Cd	As	Pb	Cr
Total		1.82E-06	1.58E-06	3.87E-05	1.79E-05	7.47E-05
Areas	Urban	2.09E-06	1.46E-06	2.94E-05	1.14E-05	5.57E-05
	Rural	1.53E-06	1.71E-06	4.85E-05	2.48E-05	9.48E-05
Sex	Men	1.81E-06	1.58E-06	3.77E-05	1.78E-05	7.37E-05
	Women	1.53E-06	1.52E-06	3.34E-05	1.57E-05	5.69E-05
Age	18–44	2.14E-06	1.68E-06	4.11E-05	1.94E-05	9.03E-05
	45–59	2.09E-06	1.46E-06	2.94E-05	1.14E-05	5.57E-05
	60–	1.53E-06	1.71E-06	4.85E-05	2.48E-05	9.48E-05

### Exposure levels of Hg, Cd, As, Pb, and Cr through air

The total exposures of heavy metals through air were 2.18 × 10^−9^ mg/(kg·d), 2.18 × 10^−8^ mg/(kg·d), 5.22 × 10^−7^ mg/(kg·d), 4.46 × 10^−7^ mg/(kg·d) and 7.61 × 10^−7^ mg/(kg·d) for Hg, Cd, As, Pb, and Cr, respectively. The highest exposure levels of Hg, Cd, As, Pb, and Cr were found in Chengdu, Dalian, Lanzhou, Wuhan, and Dalian, respectively, while the lowest exposure levels were found in Lanzhou, Shanghai, Taiyuan, Shanghai, and Lanzhou, respectively. The concentration and comprehensive exposure coefficient of air were higher in urban areas, leading to a significant difference (p < 0.05) between the exposure levels of heavy metals in urban areas compared to rural areas. The exposure level of heavy metals through the air can be broken down into three components: indoor air, outdoor air, and traffic air. These three components can be thought to contribute to exposure levels through air in the order of indoor air > outdoor air > traffic air because people generally spend a long time indoors (approximately 1,168 minutes per day)^22,23^.

### Heavy metal exposure through indoor air

**Figure 2:**
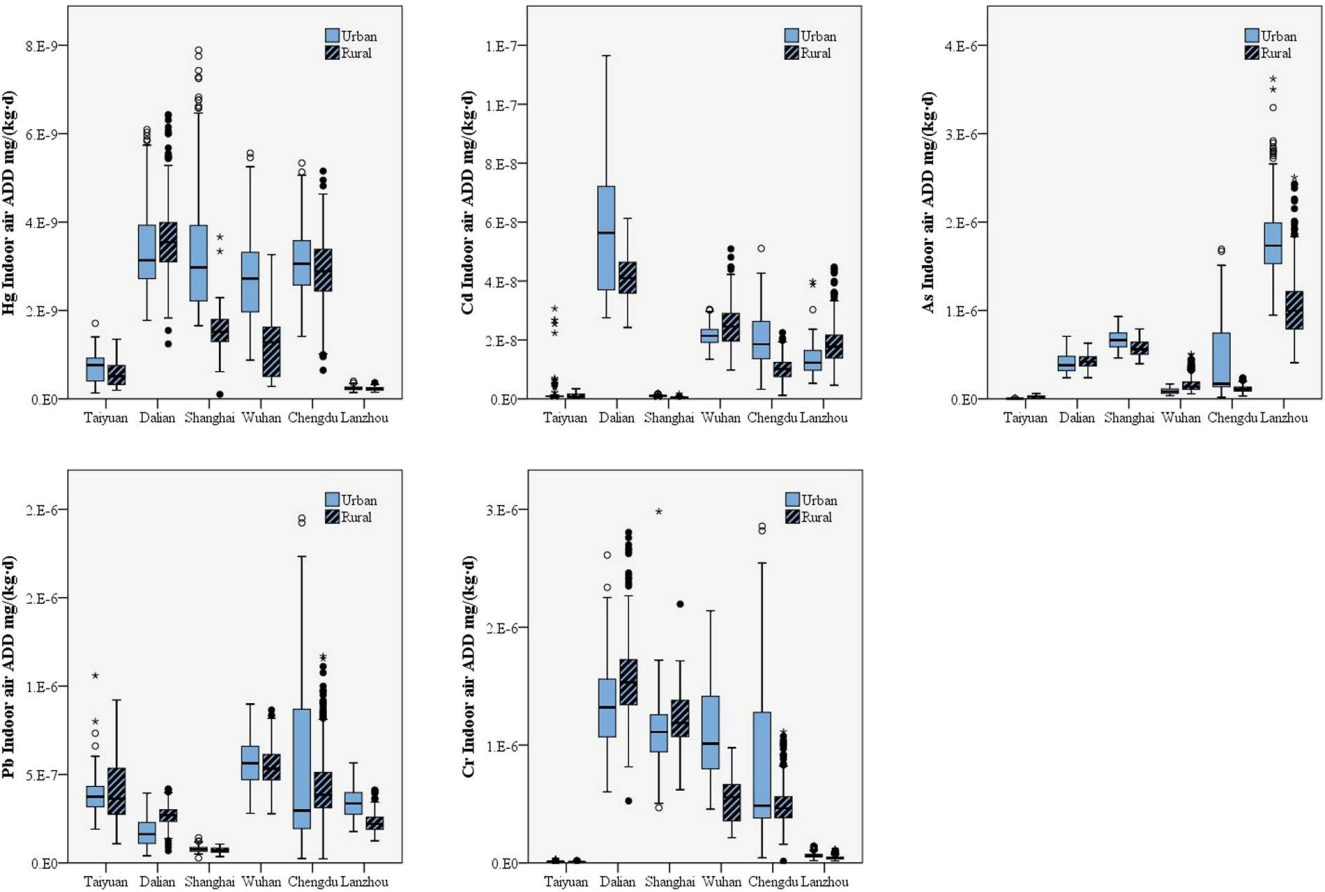
Exposure levels for Hg, Cd, As, Pb and Cr through indoor air in the six areas under study.

The exposure levels of the five heavy metals from indoor air samples decreased in the order of Cr > As > Pb > Cd > Hg (Fig. 2). There was a significant difference (p < 0.01) in the exposure level of heavy metals in the indoor air samples among the six cities. Dalian was shown to have the most severe exposure to Hg, Cd and Cr, while Lanzhou had the highest As exposure level and Wuhan had the highest exposure level of Pb. Dalian had a lower comprehensive exposure coefficient for indoor air, but the highest concentrations of Hg, Cd and Cr led to the highest exposure of the six cities tested. In contrast, although the comprehensive exposure coefficients for Hg, Cd and Cr was not the highest in Lanzhou, a large concentration of As contributed to the considerable exposure level for the city. Overall, urban areas showed higher exposure levels from indoor air than rural areas due to the longer time spent indoors by urban residents.

The exposure levels of the different sexes through indoor air were significantly different (p < 0.05), and men were shown to be exposed more than women because the comprehensive exposure coefficient for indoor air for men was higher than that for women (0.1915 m^3^/(kg·d) for men vs. 0.1576 m^3^/(kg·d) for women). The difference in exposure levels among the three age groups was significant as well (p < 0.05), and the exposure order was 18 – 44 years old > 45–59 years old > over 60 years old, consistent with the comprehensive exposure coefficients. There are many factors that could affect the indoor air exposure level, such as cooking fuel, types of floor covering, and heating patterns. For example, the exposure of As in indoor air for residents using biomass, coal, gas, and electricity as cooking fuel were 1.87 × 10^−9^ mg/(kg·d), 1.77 × 10^−9^ mg/(kg·d), 1.39 × 10^−9^ mg/(kg·d), and 1.29 × 10^−9^ mg/(kg·d), respectively; biomass was easily recognizable as the primary contributor to As exposure in indoor air. In Wuhan, residents responded via questionnaire that the proportion of solid fuels was 48.30% in rural areas, compared to 0% in urban areas. This can be a contributing factor to the relatively high exposure levels of As from indoor air in this region^24–26^.

### Heavy metal exposure through outdoor air

**Figure 3:**
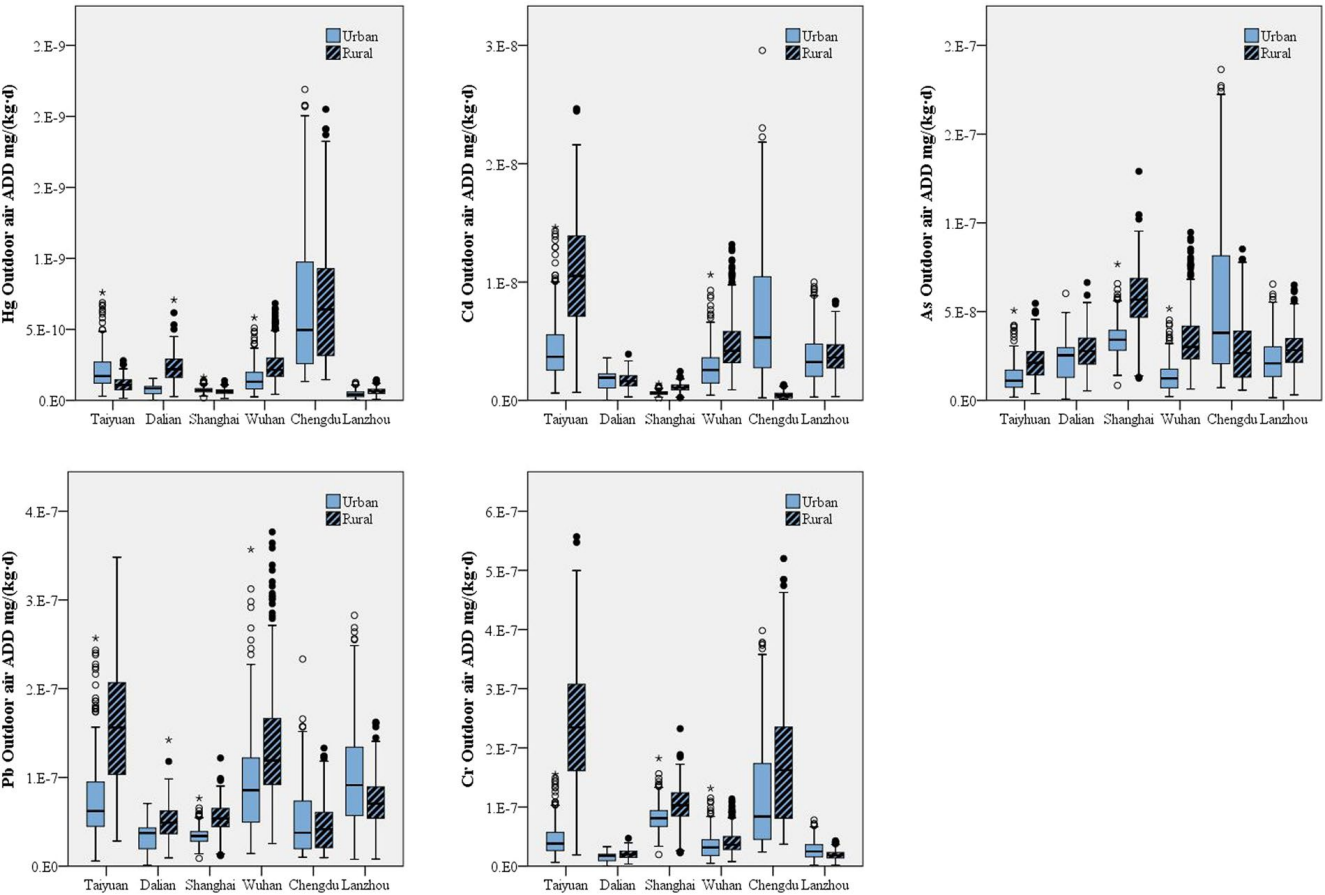
Exposure levels for Hg, Cd, As, Pb and Cr through outdoor air in the six areas under study.

The heavy metal exposure from outdoor air decreased in the order of Pb > Cr > As > Cd > Hg (Fig. 3). The content of the metals varied in the different cities (p < 0.05). Hg, Cd, As, Pb, and Cr had the highest exposure levels in Chengdu, Taiyuan, Shanghai, Taiyuan and Chengdu, respectively, while the lowest exposure levels were found in Lanzhou, Shanghai, Taiyuan, Dalian, and Dalian, respectively. Compared to indoor air, outdoor air was found to have less heavy metal pollutants, which can be explained by the higher bioactivity and migratory aptitude of the heavy metals found in indoor air^27^. Men were found to have received higher exposure than women (p < 0.05) because of their larger comprehensive exposure coefficient. The volunteers over 60 received lower exposures because of their smaller exposure coefficient compared to the rest of the age groups in the study.

### Heavy metal exposure through traffic air

**Figure 4:**
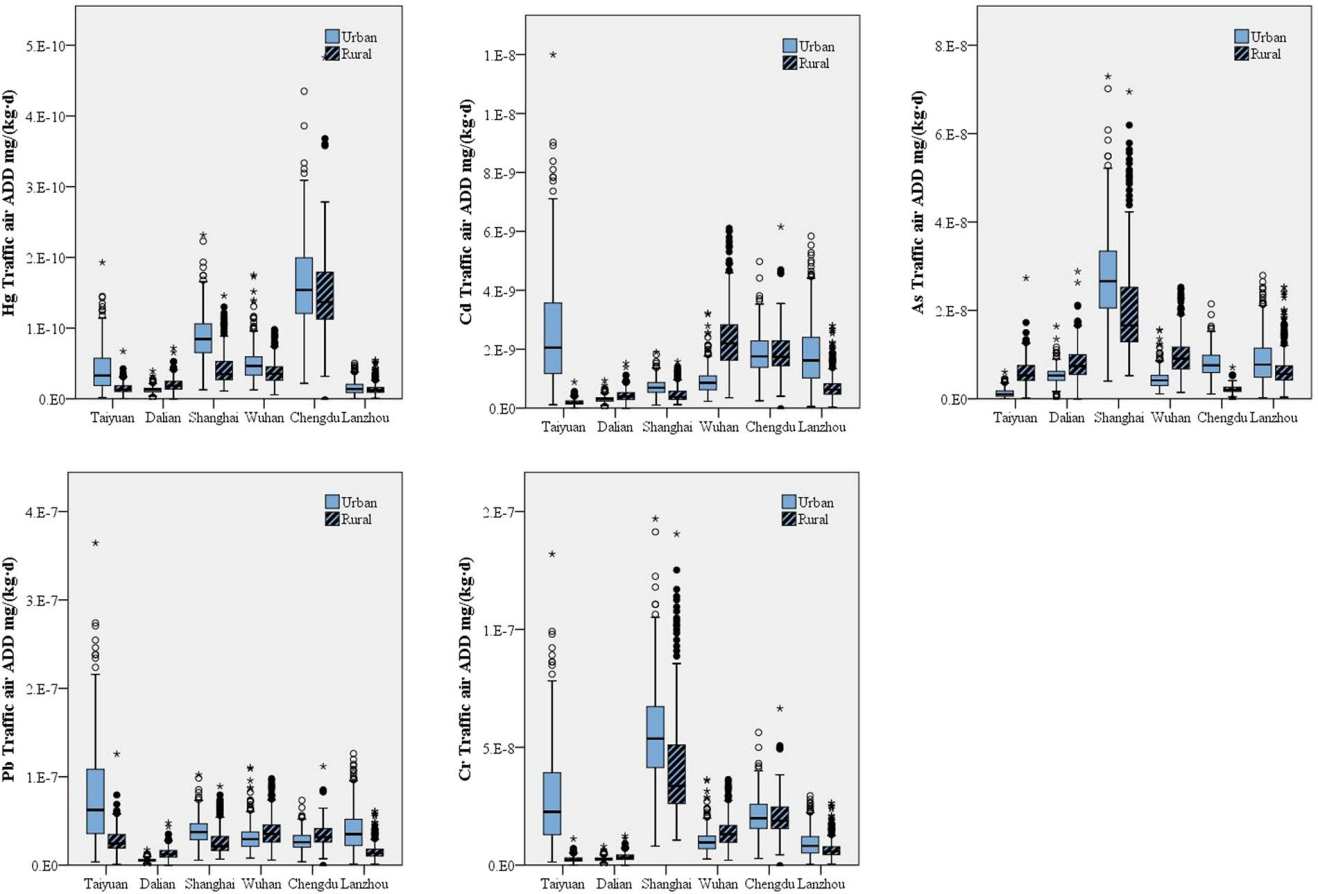
Exposure levels for Hg, Cd, As, Pb and Cr through traffic air in the six areas under study.

The exposure levels of the five heavy metals under examination from traffic air decreased in the order of Pb > Cr > As > Cd > Hg (Fig. 4). There was regional disparity among the six tested areas, and the differences in exposure levels between urban and rural areas were significant (p < 0.05). Shanghai had the highest exposure levels of As and Cr. Chengdu had the highest exposure levels of Cd and Hg. Taiyuan had the highest exposure level of Pb. Dalian showed the lowest exposure levels of Cd, Pb, and Cr, while Lanzhou had the lowest exposure level of Hg. The As exposure level was lowest in Taiyuan. Overall, urban areas showed higher exposure levels because of their larger comprehensive exposure coefficient for traffic air than rural areas (0.0103 m^3^/(kg·d) in urban areas vs. 0.0091 m^3^/(kg·d) in rural areas). Women were exposed to less heavy metals than men, which might be due to their smaller exposure coefficient for traffic air (0.0111 m^3^/(kg·d) for men vs. 0.0084 m^3^/(kg·d) for women) and shorter daily travel time (1.1136 h/d for men vs. 1.0425 h/d for women). The exposure level of residents aged 18–44 years old was the highest, while those over 60 years old had the lowest heavy metal exposure.

### Heavy metal exposure through water

**Figure 5:**
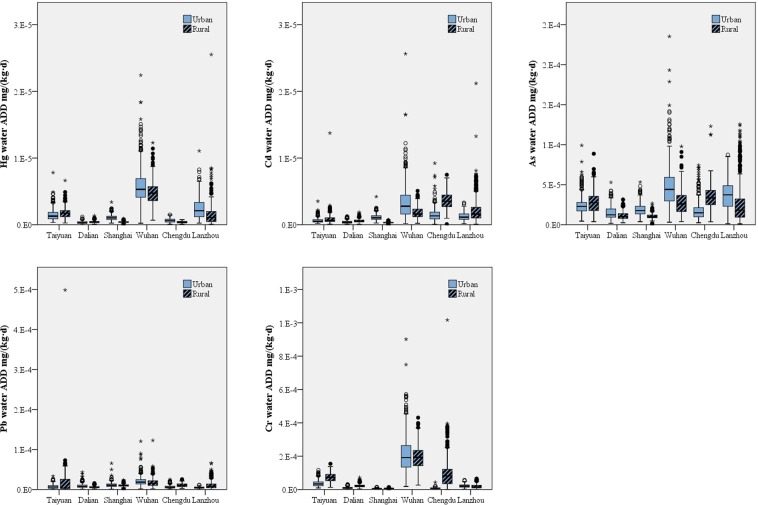
Exposure levels for Hg, Cd, As, Pb and Cr through water in the six areas under study.

Heavy metal exposure from water decreased in the order of Cr > As > Pb > Hg > Cd (Fig. 5). The six tested areas showed a significant difference in the exposure levels (p < 0.05). Wuhan had much higher exposure levels of all five metals in water compared to the other five areas. Dalian showed the lowest exposure levels of Hg, Cd, As, and Pb, while the lowest exposure level of Cr was found in Shanghai. In general, rural areas were more exposed to Cd, Pb and Cr than urban areas, but the contrary was found to be true for Hg and As. The exposure levels of As and Hg for women were significantly higher than for men (p < 0.05) due to their larger comprehensive exposure coefficients for drinking and using water, which was different to the exposure through air (men: 0.0229 L/(kg·d) for drinking water and 0.0032 m^2^/(kg·d) for using water; women: 0.0241 L/(kg·d) for drinking water and 0.0035 m^2^/(kg·d) for using water). Residents over 60 years old had the highest heavy metal exposure from water, while residents between 18 and 44 years old had the lowest exposure (p < 0.05). The comprehensive exposure coefficient for water was not the only important factor in determining heavy metal exposure; the type of drinking water can also influence exposure levels. For instance, the well and pit water used in Lanzhou had higher Hg concentrations compared to the centralized water supply used elsewhere, leading to the higher exposure level of Hg from water in Lanzhou.

### Heavy metal exposure through soil

**Figure 6:**
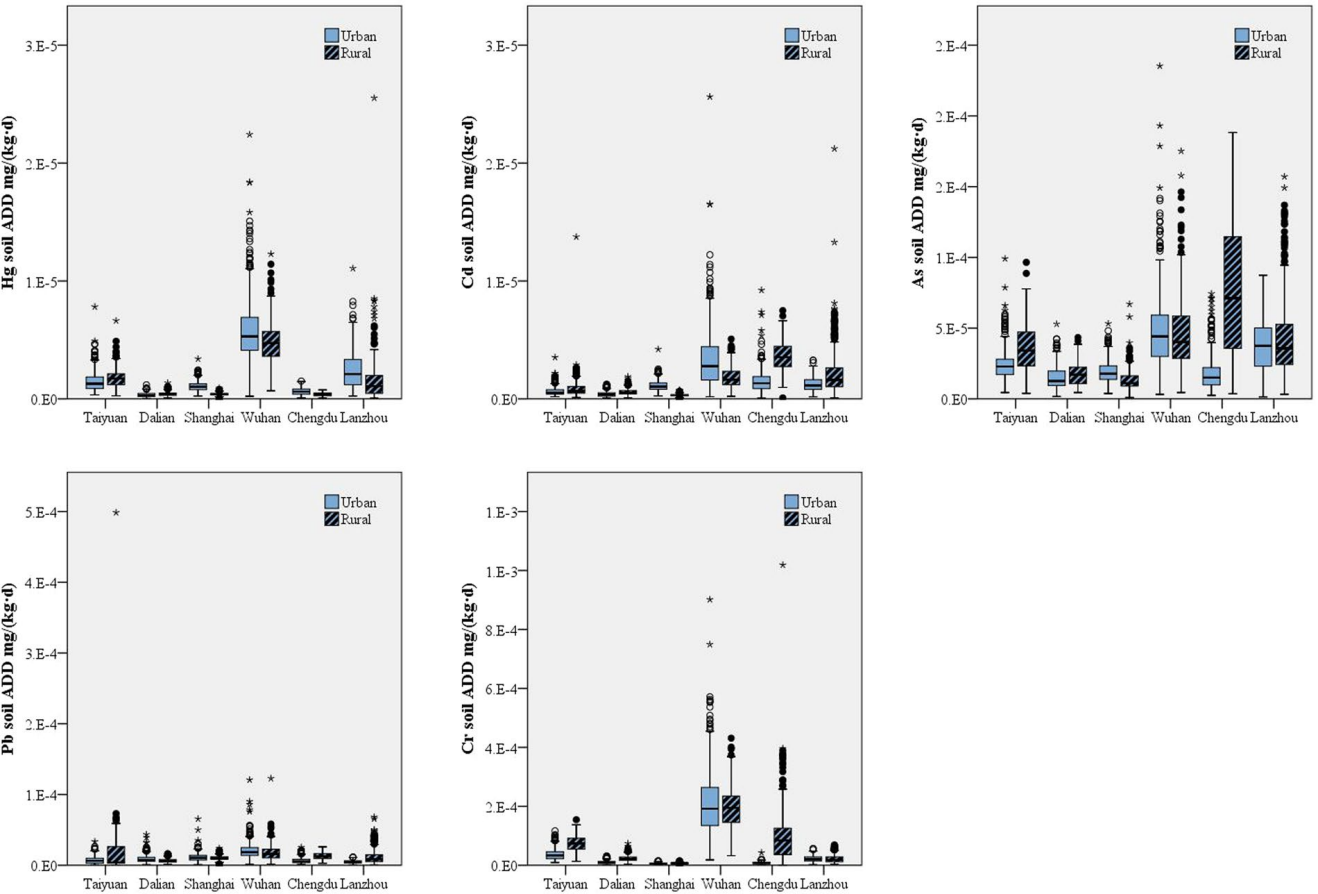
Exposure levels for Hg, Cd, As, Pb and Cr through soil in the six areas under study.

The exposure levels of the five heavy metals through soil decreased in the order Cr > As > Pb > Cd > Hg (Fig. 6), consistent with their abundance in Earth’s crust^28^. Exposure levels through soil showed a significant difference among the six areas (p < 0.05). Wuhan showed the highest soil exposure levels of Hg and Cr. Chengdu showed the highest exposure levels of Cd, As, and Pb from soil samples. Hg, Cd, As, Pb, and Cr were found to have the lowest exposure levels in Dalian, Shanghai, Dalian, Shanghai and Taiyuan, respectively. Rural volunteers were found to have higher soil exposures (p < 0.05) because they have longer contact times with soil and a higher proportion of respondents who are in contact with soil on a regular basis. The soil contact time was 1.27 h/d in urban areas and 4.57 h/d in rural areas, while the contact proportions were 3.03% in urban areas and 51.04% in rural areas. Men’s higher exposure level from soil can be ascribed to longer contact time with soil (4.61 h/d for men vs. 4.11 h/d for women, who regularly contact with soil). Residents between 45 and 59 years old were found to have the highest heavy metal exposures from soil, while residents between 18 and 44 years old had the lowest soil exposure.

### Exposure contributions of different media

**Figure 7:**
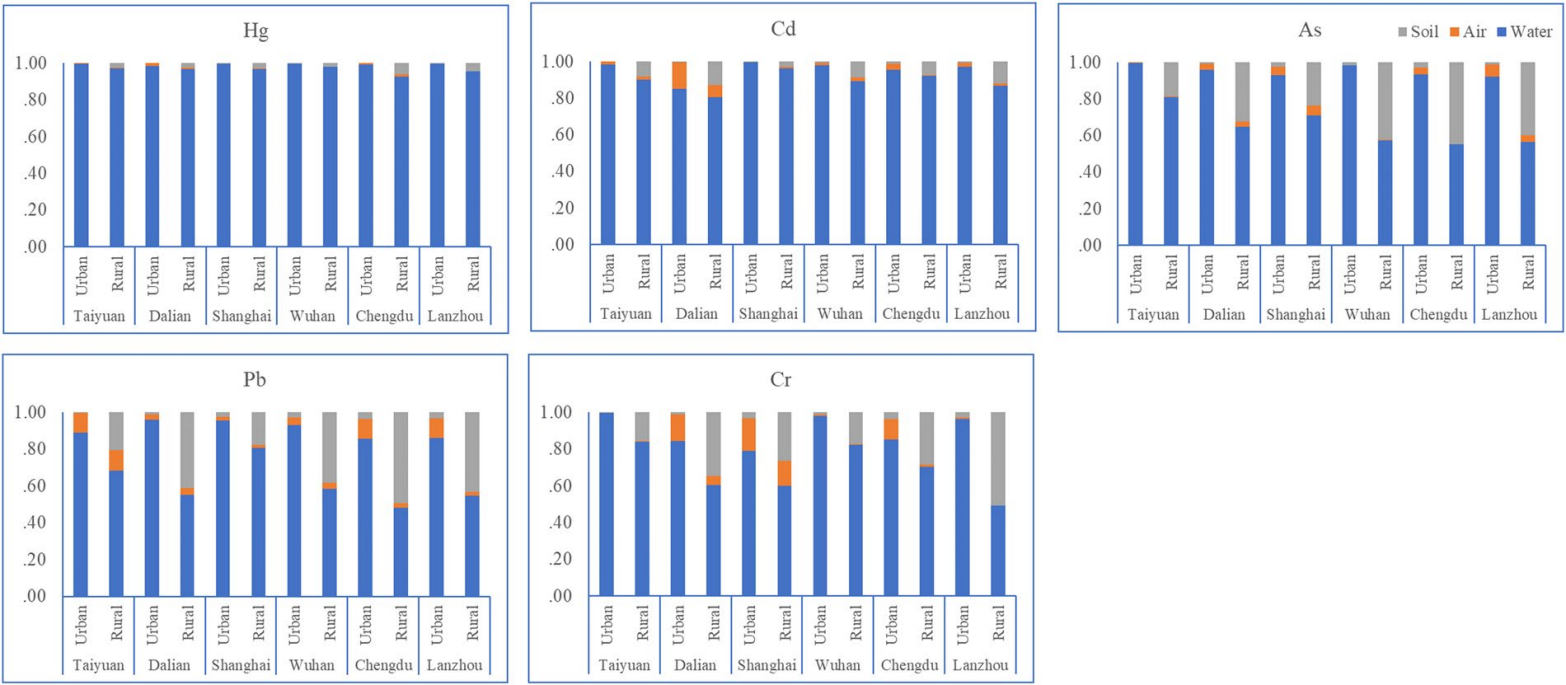
Exposure contribution of Hg, Cd, As, Pb and Cr in the six areas under study.

The heavy metals in water contributed more to the total exposure levels of Hg, Cd, As, Pb, and Cr among the environmental media tested (Fig. 7). The exposure contributions of water were 97.87%, 92.50%, 80.51%, 76.16% and 79.46% for Hg, Cd, As, Pb, and Cr, respectively. The exposure contributions of soil were 1.71%, 4.61%, 16.90%, 18.13% and 15.34% for Hg, Cd, As, Pb, and Cr, while the exposure contributions of air were 0.42%, 2.90%, 2.60%, 5.71% and 5.20%, respectively. The difference in exposure levels between urban and rural areas was significant (p < 0.05). Water was the primary contributor to total exposure in both urban and rural areas, but the proportions were different. The exposure contributions of Hg, Cd, As, Pb, and Cr from water were 99.45%, 95.87%, 95.55%, 90.61% and 91.03% in urban areas, while they were 96.20%, 88.92%, 64.59%, 60.87% and 67.21% in rural areas, respectively. In urban areas, the exposure contributions of the media decreased in the order of water > air > soil. The contributions decrease in the order of water > soil > air in rural areas due to longer soil contact times (1.27 h/d in urban areas vs. 4.57 h/d in rural areas), a higher proportion of respondents who regularly come into contact with soil (3.03% in urban areas vs. 51.04% in rural areas), and larger comprehensive exposure coefficients for soil through ingestion and dermal contact in rural areas. With respect to sex and age, there was a consistent pattern in which the exposure contributions ranked in the order of water > soil > air.

It has been shown that water was the most important contributor to the total human environmental exposure of heavy metals. This is in accordance with studies conducted in Japan in 2003^17^ and America in 2002^18^. In addition, water was found to be the largest contributor in this study, and other studies have revealed that soil (England in 2000^29^) and air (a 7 city study in Korea from 1993–2003^30^) can play the most important role in heavy metal exposure levels in other locations around the world.

## Conclusions

The total human environmental exposures of Hg, Cd, As, Pb, and Cr were 1.82 × 10^−6^ mg/(kg·d), 1.58 × 10^−6^ mg/(kg·d), 3.87 × 10^−5^ mg/(kg·d), 1.79 × 10^−5^ and 7.47 × 10^-^5 mg/(kg·d), respectively. Water was the main contributor to total exposure. The average contributions of air, water and soil ranged from 0.42% ~ 5.71%, 76.16% ~ 97.87% and 1.71% ~ 18.13%, respectively. There were differences in the exposure contribution of urban and rural areas. The exposure contribution decreased in the order of water > air > soil and water > soil > air in urban and rural areas, respectively. The high proportion of soil contact and longer contact time in rural areas accounted for the change in the order of soil and air in rural areas. Therefore, residents in rural areas should pay more attention to protecting themselves against soil exposure to heavy metals.

The average daily exposure levels of the five heavy metals in different environmental media had different trends. The exposure level of Cr was the highest of the heavy metals tested, except for outdoor and traffic air samples. The exposure levels of Hg were lowest in all the media, except for water. As far as air is concerned, the total exposure levels of five heavy metals through air, overall, were consistent with the exposure level of indoor air, which had the highest levels of Cr and the lowest Hg exposure. Indoor air exposure accounted for more than 70% of the total air exposure levels, which was caused by higher indoor activity times. Outdoor air and traffic air exposure levels had the highest Pb exposure and the lowest Hg exposure. The indoor air exposure levels were not consistent with the outdoor air exposure levels for the different metals. The influence of air infiltration factors, as well as the complex and diverse sources of pollutants in the room, were the main factors causing the inconsistency in these indoor and outdoor air samples. Special attention needs to be paid to the prevention and control of air pollution health risks.

There were regional, urban-rural, sex, and age differences between the total exposure levels of Hg, Cd, As, Pb, and Cr, which are related to differences in the natural environment, society, economy, and lifestyle, among other factors. Behaviour patterns and environmental quality both affect the total exposure levels, but behaviour patterns are the main influencing factors within the same region. The environmental qualities of different regions are the main influencing factors of heavy metal exposure. At the same exposure concentration, the exposure levels of different sexes and different age groups are closely related to the exposure behaviour patterns of each environmental medium.

This study offers a great contribution by filling gaps in the total exposure field. Distinguishing features of the total exposure of heavy metals in different areas are identified, showing exposure levels and contribution rates of variable media, which can offer support for environmental health criteria, as well as help to confirm the needed emphasis on risk control for public health.

## Materials and Methods

### Study sites

After comprehensive analysis of the location, industrial structure, climate, and ecological environment, six areas of China were chosen, taking into account the environmental exposure-related human time-activity patterns for these areas. Taiyuan, Dalian, Shanghai, Wuhan, Chengdu and Lanzhou are located in Northern China, Northeast China, Eastern China, Southern China, Southwest China, and Northeast China, respectively.

### Questionnaire survey method

The protocols used were approved by Institutional Review Boards (IRB) at the Tongji Medical College of Huazhong University of Science and Technology, and informed consent forms were obtained from all volunteers. Adults aged 18 years or older were randomly selected from the six cities. Participants had been living at the survey site for more than six months without a history of long-term medication use. A face-to-face survey method was used to obtain important factors influencing the environmental exposure of heavy metals, including intake/ingestion factors, time-activity factors, physical (body weight, skin surface area, inhalation rate) factors and other environment-related (heating duration) factors.

A total of 3,855 volunteers took part in our study from urban and rural areas of the six chosen areas from October 2016 to May 2017. There were 1,982 urban residents and 1,873 rural residents, and there were 1,862 men and 1,993 women. The age distribution included 1,587 residents between 18 and 44 years old, 1,119 residents between 45 and 59 years old and 1,149 residents over 60 years old.

### Sampling methods

The water, soil, and air exposure samples were collected from 10% of the volunteers selected randomly. A total of 2,939 samples were collected, and the distribution of the samples can be found in Table 3. Methods for sampling, pretreatment, and analysis were performed in accordance with the relevant guidelines and regulations.

**Table 3:** Sample distribution in this study.

Area		Total	Exposure media				
			Air			Water	Soil
			Indoor	Outdoor	Traffic		
Total		2939	583	874	271	1065	146
Taiyuan	Total	492	115	144	48	161	24
	Urban	245	55	72	24	82	12
	Rural	247	60	72	24	79	12
Dalian	Total	319	72	71	35	120	21
	Urban	142	28	24	21	58	11
	Rural	177	44	47	14	62	10
Shanghai	Total	501	93	156	62	163	27
	Urban	272	50	71	37	97	17
	Rural	229	43	85	25	66	10
Wuhan	Total	616	120	234	48	190	24
	Urban	333	65	132	24	100	12
	Rural	283	55	102	24	90	12
Chengdu	Total	463	103	143	42	139	36
	Urban	248	53	72	30	75	18
	Rural	215	50	71	12	64	18
Lanzhou	Total	548	80	126	36	292	14
	Urban	314	40	90	18	156	10
	Rural	234	40	36	18	136	4

We used intelligent volume samplers (TH-150, Wuhan Tianhong Intelligent Instrument Factory, Wuhan, China) to collect the outdoor and traffic air samples. The indoor air samples were collected by personal air samplers (BUCK LP-5, A.P. BUCK INC. USA). Quartz filters (37 mm and 90 mm diameter, Whatman Inc., USA) were used as the filter membranes in the air samplers. To remove volatile substances and other impurities, the filter membranes were baked at 400 °C for 6 hours prior to sampling. A scale that is accurate to one hundred-thousandth of a gram (XPR56/AC, METTLER TOLEDO, Switzerland) was used for all weighing purposes. Indoor air sampling was conducted once every three days, and each sample was obtained by continuous sampling for at least 60 hours in the 3-day period. Outdoor and traffic air sampling was conducted once per day, and each sample was obtained by sampling for no less than 20 hours in that day and the corresponding 3 days. A total of 1,728 filter membranes were collected among the indoor, outdoor, and traffic air samples. All samples were sealed in tinfoil pouches and kept at 4 °C before the heavy metals trapped in the filter membranes were analysed, as described below.

Water samples were obtained from the end of tap waterpipes or from containers, such as water vat, representing drinking and using water with high frequency and large intake. All samples were collected in high density polyethylene bottles, which had been washed with 20% HNO_3_. Water samples were stored at 4 °C with HNO_3_ preservative.

Topsoil samples from the top 0–20 cm and weighing approximately 1 kg each, were collected at 4–5 sub-sampling sites; the samples were obtained by the quartering method after removing impurities.

### Sample analysis

Each air sample filter membrane was completely digested with HNO_3_-HCl before further analysis^31^. Water samples were filtered through 0.45 μm membranes. Dry soil samples were ground in an agate mortar and then sieved (100 mesh, 0.149 pore diameter) and finally digested with HNO_3_-HCl before analysis. The concentrations of Cd, Pb, and Cr were identified using ICP-MS (Thermo Fisher Scientific, America), while the concentrations of Hg and As were identified using atomic fluorescence spectrometry. Certified reference materials and reagent blanks were used for quality control in the sample analysis. Standard addition was applied to samples without certified reference materials, and the recoveries obtained using this method were acceptable.

### Exposure assessment

The average daily dose (ADD) (mg/(kg·day)) of an element though air, water, soil, and diet was estimated using Eqs (1)–(9)^32–34^:

1 $${{\rm{ADD}}}_{{\rm{total}}}={{\rm{ADD}}}_{{\rm{air}}}+{{\rm{ADD}}}_{{\rm{water}}}+{{\rm{ADD}}}_{{\rm{soil}}}$$

2 $${{\rm{ADD}}}_{{\rm{air}}}=\frac{{{\rm{C}}}_{a}\times {\rm{InhR}}\times {\rm{ET}}\times {\rm{EF}}\times {\rm{ED}}}{{\rm{BW}}\times {\rm{AT}}}$$

3 $${{\rm{ADD}}}_{{\rm{water}}}={{\rm{ADD}}}_{{\rm{w}} \mbox{-} {\rm{oral}}}+{{\rm{ADD}}}_{{\rm{w}} \mbox{-} {\rm{dermal}}}$$

4 $${{\rm{ADD}}}_{{\rm{w}} \mbox{-} {\rm{oral}}}=\frac{{{\rm{C}}}_{{\rm{w}}}\times {\rm{Ing}}{{\rm{R}}}_{{\rm{w}}}\times {\rm{EF}}\times {\rm{ED}}}{{\rm{BW}}\times {\rm{AT}}}$$

5 $${{\rm{ADD}}}_{{\rm{w}} \mbox{-} {\rm{dermal}}}=\frac{{{\rm{C}}}_{{\rm{w}}}\times {{\rm{SA}}}_{{\rm{w}}}\times {\rm{PC}}\times {{\rm{CF}}}_{{\rm{w}}}\times {\rm{ET}}\times {\rm{EF}}\times \mathrm{ED}\,}{{\rm{BW}}\times {\rm{AT}}}$$

6 $${{\rm{ADD}}}_{{\rm{soil}}}={{\rm{ADD}}}_{{\rm{s}} \mbox{-} {\rm{dermal}}}+{{\rm{ADD}}}_{{\rm{s}} \mbox{-} {\rm{oral}}}+{{\rm{ADD}}}_{{\rm{s}} \mbox{-} {\rm{inh}}}$$

7 $${{\rm{ADD}}}_{{\rm{s}} \mbox{-} {\rm{inh}}}=\frac{{{\rm{C}}}_{{\rm{s}}}\times {\rm{InhR}}\times {\rm{EF}}\times {\rm{ED}}}{{\rm{PEF}}\times {\rm{BW}}\times {\rm{AT}}}$$

8 $${{\rm{ADD}}}_{{\rm{s}} \mbox{-} {\rm{oral}}}=\frac{{{\rm{C}}}_{{\rm{s}}}\times {\rm{Ing}}{{\rm{R}}}_{{\rm{s}}}\times {{\rm{CF}}}_{{\rm{s}}}\times {\rm{FI}}\times {\rm{EF}}\times {\rm{ED}}}{\mathrm{BW}\times \mathrm{AT}}$$

9 $${{\rm{ADD}}}_{{\rm{s}} \mbox{-} {\rm{dermal}}}=\frac{{{\rm{C}}}_{{\rm{s}}}\times {\rm{CF}}\times {{\rm{SA}}}_{{\rm{s}}}\times {\rm{AF}}\times {{\rm{ABS}}}_{{\rm{d}}}\times {\rm{EF}}\times {\rm{ED}}}{{\rm{BW}}\times {\rm{AT}}}$$

where ADD_total_ is the sum of the average daily doses (mg/(kg·d)) of air, water and soil, ADD_air_ is the average daily dose (mg/(kg·d)) of an element via the inhalation of air, C_a_ is the concentration of the element in the air (mg/m^3^), InhR is the inhalation rate (m^3^/h), ET is the exposure time (h/d), EF is the exposure frequency (d/year), ED is the exposure duration (years), BW is the body weight (kg), AT is the average time (days), ADD_water_ is the average daily dose (mg/(kg·d)) of an element in water, ADD_w-oral_ is the average daily dose (mg/(kg·d)) of an element via the ingestion of water, ADD_w-dermal_ is the average daily dose (mg/(kg·d)) of an element through dermal contact of water, C_w_ is the concentration of the element in water (mg/L), IngR_w_ is the ingestion rate of water (L/d), SA_w_ is the surface area of the skin exposed to the pollutants in water (cm^2^), CF_w_ is the volume conversion factor (1 L/1000 cm^3^), ADD_soil_ is the average daily dose (mg/(kg·d)) of an element in soil, ADD_s-dermal_ is the average daily dose (mg/(kg·d)) of an element via dermal contact with soil, ADD_s-oral_ is the average daily dose (mg/(kg·d)) of an element via the ingestion of soil, ADD_s-inh_ is the average daily dose (mg/(kg·d)) of an element via inhalation of soil, C_s_ is the concentration of the element in the soil (mg/kg), PEF is the particulate emission factor (m^3^/kg), IngR_s_ is the ingestion rate of soil (mg/d), CF_s_ is the conversion factor (10^−6^), FI is the digestive tract absorption factor, SA_s_ is the surface area of the skin exposed to pollutants of soil (cm^2^·event^−1^), AF is the skin adherence factor (mg/cm^2^), and ABS_d_ is the dermal absorption factor. InhR, IngR_w_, IngR_s_, SA_w_, SA_s_, BW and activity-time pattern factors were obtained through volunteer responses to the questionnaire. The other exposure parameters were obtained from the literature and are listed in Table 4^23,34–37^.

**Table 4:** Parameters and their reference values.

Parameter	Reference value
Inhalation rate (InhR)	From questionnaire
Ingestion rate of water (IngRw)	From questionnaire
Ingestion rate of soil (IngRs)	From questionnaire
Surface area of the skin exposed to pollutants of water (SAw)	From questionnaire
Exposure duration (ED)	From questionnaire
Exposure time (ET)	From questionnaire
Body weight (BW)	From questionnaire
Exposed skin area (SA)	From questionnaire
Conversion factor (CFs)	10^−6^
Digestive tract absorption factor (FI)	When calculating the risk we need to use 0.07 for Hg, 0.025 for Cd, Pb and Cr
Volume conversion factor (CFw)	1 L/1000 cm^3^
Exposure frequency (EF)	365 d/year
Averaging time (AT)	For non-cancer, AT = ED × 365; for cancer, AT = 74.8 × 365
Particle emission factor (PEF)	1.36 × 10^9^ m^3^/kg
Skin adherence factor (AF)	0.2
Dermal absorption factor (ABS_d_)	0.001 for Hg, Cd, Pb and Cr, 0.03 for As

### Statistical analysis

Questionnaires were input with an investigator-developed online system by a face-to-face method and were confirmed by a quality controller. Data cleaning and the related analyses were performed with JMP14 software. The questionnaire data cleaning was based on key variables, such as height, weight, and age, and included repairing the missing data and deleting the abnormal data. The mean and median values of exposure levels were calculated. To account for the complex sampling design in the investigation, a significance test was performed.

## Supplementary information

Database 1
